# Current Treatment Options for Chronic Myeloid Leukemia Patients Failing Second-Generation Tyrosine Kinase Inhibitors

**DOI:** 10.3390/jcm9072251

**Published:** 2020-07-15

**Authors:** Valentín García-Gutiérrez, Juan Carlos Hernández-Boluda

**Affiliations:** 1 Hematology Service, Hospital Universitario Ramón y Cajal, IRYCIS, 28034 Madrid, Spain; 2 Hematology Service, Hospital Clínico Universitario INCLIVA, 46010 Valencia, Spain; hernandez_jca@gva.es

**Keywords:** chronic myeloid leukemia, second-generation tyrosin kinase inhibitors, treatment, safety, efficacy

## Abstract

Despite the excellent overall survival (OS) of patients with chronic myeloid leukemia (CML), a significant proportion will not achieve optimal response to imatinib or second-generation tyrosine kinase inhibitors (2GTKI). For patients with inadequate response to 2GTKIs, alternative 2GTKIs or ponatinib are widely available treatment options in daily clinical practice. Treatment decisions should be guided by correct identification of the cause of treatment failure and accurate distinction between resistant from intolerant or nonadherence patients. This review aims to provide practical advice on how to select the best treatment option in each clinical scenario.

## 1. Introduction

The prognosis of chronic myeloid leukemia (CML) has improved dramatically over the last few decades, moving from a disease with a median overall survival (OS) of 3 years to a new scenario in which most patients enjoy similar OS to the overall population [1]. Clearly, the main reason for such an improvement has been the addition of several tyrosine kinase inhibitors (TKIs) into the clinic [1,2]. Currently, five TKIs are widely available for use in daily clinical practice. They have differing efficacy and tolerability profiles, of which attending physicians should be aware for optimal treatment selection in each situation. Imatinib and three second-generation TKIs (2GTKIs; nilotinib, dasatinib, and bosutinib) are approved in the first-line setting, of which overall 2GTKIs have shown higher rates of deep molecular responses that could increase the probability of treatment discontinuation. Although no differences in long-term OS have been observed among TKIs, 2GTKI are usually the preferred option for high-risk CML patients [3].

On the other hand, a significant proportion of patients (approximately 40%) will discontinue first-line TKI treatment due to toxicity or lack of efficacy, regardless of TKI type [4,5,6]. Similar efficacy has been documented with all 2GTKIs after failed imatinib, with 40% and 60% probabilities of optimal response in imatinib resistant and intolerant patients, respectively. Of note, approved nilotinib and bosutinib doses are higher in the second-line setting than in front-line [7,8,9].

Management of patients failing 2GTKI is currently challenging, due to the limited data on different therapies from comparative studies. This review will examine the evidence regarding treatment options for CML patients failing 2GTKI, including new drugs currently being tested in clinical trials.

## 2. Approved Treatment Options

### 2.1. Dasatinib and Nilotinib

Dasatinib and nilotinib have been broadly used to rescue patients after 2GTKI failure in the absence of other available treatments besides stem cell transplantation [10,11,12]. However, there still a paucity of clinical data on the results of this treatment approach [13].

Use of nilotinib as a third-line treatment in patients failing imatinib and dasatinib was evaluated in a phase II clinical trial including recruiting 37 CML patients in chronic phase (CP). After 12 months of treatment, 24% achieved complete cytogenetic response (CCyR), with only 54% remaining on the study [14]. Dasatinib treatment has not been prospectively evaluated in patients with prior failed response to nilotinib.

Several studies have evaluated dasatinib and nilotinib as a third-line therapy in routine clinical practice. Overall, CCyR rates around 10–35% were reported, with higher response rates in intolerant than resistant patients [10,11,12].

### 2.2. Bosutinib

Bosutinib is a dual Src/Abl TKI with minimal inhibitory activity against c-KIT and platelet-derived growth factor receptor (PDGFR), the two nonspecific targets potentially associated with toxicities with other 2GTKIs. Bosutinib has demonstrated significant clinical activity in patients with CP CML who had resistance or intolerance to prior TKI therapy [15].

Bosutinib, at a dose of 500 mg QD, was first evaluated in a phase I/II clinical trial in CML patients with at least two failed TKIs. Overall, in the setting of third- and fourth-line therapy, cumulative rates of confirmed complete hematologic response (cCHR) and major cytogenetic response (MCyR) were 74% and 40%, respectively, including 32% patients who attained/maintained CCyR (newly attained, 26%). A total of 53 (45%) patients required dose reductions due to adverse events, with 21 of them achieving or maintaining MCyR. Ninety (76%) patients discontinued treatment by year 4, with the most common primary reasons being adverse events (24%), disease progression (20%), and lack of efficacy (18%) [16]. In a collaborative study between the Spanish CML Group (GELMC) and the Hammersmith Hospital in London, bosutinib treatment was evaluated in 62 patients with prior dasatinib and nilotinib failure. While the probabilities of obtaining CCyR and major molecular response (MMR) in resistant patients were only 25% and 14%, respectively, they increased up to 94% and 42% respectively in intolerant patients. Of interest, different dose exposure was observed in resistant and intolerant patients, with median daily dose of 500 mg and 400 mg, respectively [17].

**Figure 1:**
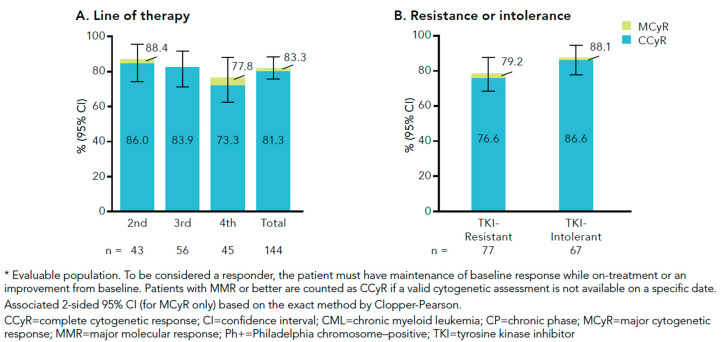
Summary of cumulative incidence of cytogenetic responses in CP CML patients enrolled in the BYOND study by (**A**) line of therapy and (**B**) resistance or intolerance to prior TKIs.

**Figure 2:**
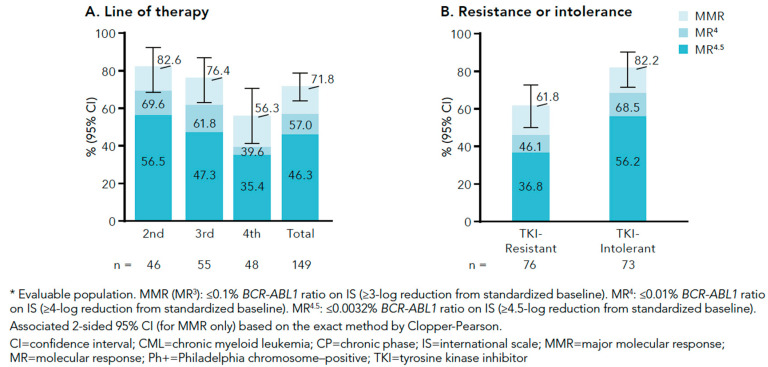
Summary of cumulative incidence of molecular response in CP CML patients enrolled in the BYOND study by (**A**) line of therapy and (**B**) resistance or intolerance to prior TKIs.

Data from a new phase IV clinical trial evaluating bosutinib in 156 CML patients failing at least one TKI have recently been presented at European and American Society conferences. The initial daily treatment dose was 500 mg, although a dose increase was allowed in case of insufficient response. The study population was stratified into three cohorts according to the corresponding treatment line: 46 patients were treated in second-line, 61 patients in third-line, and 49 patients in fourth-line. The study endpoint was confirmed major cytogenetic response (cMCyR). However, it is important to note that patients were considered responders if they newly achieved or maintained baseline MCyR for a period of 52 weeks. Among 144 evaluable patients, cMCyR at 1 year of bosutinib treatment was observed in 75% and 62% of cases after failure of 1–2 TKIs and >2 TKIs, respectively. The corresponding CCyR and MMR rates were 86% and 82%, 83% and 76%, and 73% and 56%, for patients in second-, third-, and fourth-line treatment, respectively (Figure 1 and Figure 2). As expected, response rates were dependent on treatment indication based on resistance or intolerance to previous treatments (CCyR rates of 79% vs. 88% for resistant and intolerant patients, respectively) [18]. These data are in contrast with published data from the abovementioned phase I/II trial in which significantly lower response rates were observed. Reasons for the discrepant results may be the higher number of intolerant patients in the BYOND study and differences in response criteria. Thus, BYOND study patients who maintained the baseline response were classified as responders, while in the phase I/II trial only responses achieved after bosutinib initiation were considered. A subsequent sub-analysis of BYOND was performed focusing on patients without baseline response. In this subset, CCyR rates were 65%, 50%, and 72% in second-line treatment groups after imatinib failure, 2GTKI failure, and first TKI intolerance, respectively.

Regarding safety issues, adverse events remained concurrent with those reported in previous studies. Thus, diarrhea was observed in 87% of patients (16% grade 3/4), nausea in 39% (2% grade 3/4), and hepatotoxicity in 25% (14% grade 3/4; Table 1, of which the last was the side effect most frequently leading to treatment discontinuation. After a median follow-up of 30 months, 43% of patients had abandoned treatment, mainly due to side effects (25%) and lack of efficacy (5%). No progressions to advanced stages of CML were observed during treatment and a total of 12 deaths were registered.

A total of 78% of patients required dose modification, usually leading to a reduction in side effects. Median daily dose was higher in resistant than in intolerant patients (405 mg vs. 292 mg, respectively). Cross-intolerance was considered when a patient discontinued treatment for the same side effect as had precipitated prior TKI treatment discontinuation. In general terms, cross-intolerance rates were low: 2% for prior imatinib, 7% for dasatinib, and 0% for nilotinib. It is noteworthy that this complication recurred with bosutinib in 36% of patients who had suffered pleural effusion with dasatinib, although without leading to treatment discontinuation in most cases [19].

An Italian research group recently presented the results from the Bosutinib Efficacy, Safety, Tolerability (BEST) study in elderly CML patients. A total of 63 elderly (>60 years) patients with previous failure of other TKIs were treated with bosutinib at the starting daily dose of 200 mg, with prospective dose adaptation depending on tolerability and molecular response at pre-defined time-points. Reasons for switching to bosutinib were intolerance (63%) and resistance (37%) to previous treatments. First-line TKI received was imatinib (83%), dasatinib (11%), and nilotinib (6%). After a median follow-up of 9 months, 70% of patients remained on the 300 mg daily dose, with an MMR rate of 60%. Of the 63 patients, 51 were still on bosutinib at the last contact visit: six on 400 mg, 34 on 300 mg, and 11 on 200 mg [20].

### 2.3. Ponatinib

Ponatinib is a third-generation TKI approved in patients with refractory CML or Philadelphia chromosome–positive acute lymphoblastic leukemia (Ph+ ALL), and those harboring the BCR-ABL1 T315I mutation. The PACE trial evaluated ponatinib 45 mg QD in 267 CP CML patients, of whom >90% had already received two or more TKIs. In total 56% met the primary end-point of achieving MCyR by 12 months. By 5 years follow-up, 70% of patients harboring the T315 mutation achieved CCyR. After 5 years of follow-up, 54% and 40% of patients achieved CCyR and MMR, respectively. Responses were durable, with 82% and 59% of those who achieved MCyR by 12 months and MMR at any time, respectively, maintaining response at 5 years. The number of baseline mutations did not affect survival or response rates. Unfortunately, the dose of 45 mg was associated not only with high rates of response, but also with serious side effects. Serious arterial and venous occlusive adverse events occurred in 19% and 5% of patients, respectively. Subsequent studies have demonstrated that risk of cardiovascular events during ponatinib treatment is related to patient comorbidities and ponatinib dose exposure. Dose intensity is correlated with level of response and toxicity [21,22,23]. For this reason, dose reduction is currently recommended in patients achieving optimal response to mitigate the risk of vascular events.

Different groups have evaluated ponatinib in clinical practice, including patients treated with lower ponatinib dose upfront (Table 2) [24,25,26,27,28]. Overall efficacy seems consistent with results from the PACE trial, with probabilities between 59% and 69% of obtaining CCyR in CP CML patients. Conversely, incidence of occlusive vascular events varies significantly across series. As expected, cardiovascular events were mainly associated with presence of cardiovascular risk factors, previous exposure to TKIs, and ponatinib dose exposure. Thus, incidence of vascular complications ranged from 3% in young patients without history of occlusive vascular events to 17% in a more fragile population. It is worth mentioning here that the strategy of dose decreases to mitigate toxicity has been broadly adopted in the real-world setting.

The OPTIC trial (NCT02467270) was designed to evaluate the safety and efficacy of different starting daily doses of ponatinib (15 mg, 30 mg, and 45 mg) in CML patients resistant to two prior TKIs, with predetermined drug dose reductions at specific time points. An interim analysis with a median time exposure of one year showed probabilities of obtaining BCR-ABL1 <1% of 38%, 27%, and 26% for the 45 mg, 30 mg, and 15 mg doses, respectively. Corresponding incidence of arterial occlusive events was 5%, 4%, and 1%, whereas discontinuation rates due to treatment-emerged adverse events were 18%, 15%, and 14% [29].

### 2.4. Radotinib

Radotinib is a BCR-ABL inhibitor approved in Korea for front-line treatment of CML based on results of a phase III clinical trial that demonstrated its superiority over imatinib in terms of MMR. In a phase II trial, radotinib was effective and well tolerated in patients with CP CML who did not respond to previous imatinib [30]. CCyR rate by 12 months was 47%. Overall survival (OS) and progression-free survival (PFS) rates at 12 months were 96% and 86%, respectively. However, radotinib has not been evaluated in patients failing 2GTKI [31].

## 3. Emerging Treatment Options

### 3.1. Asciminib

**Figure 3:**
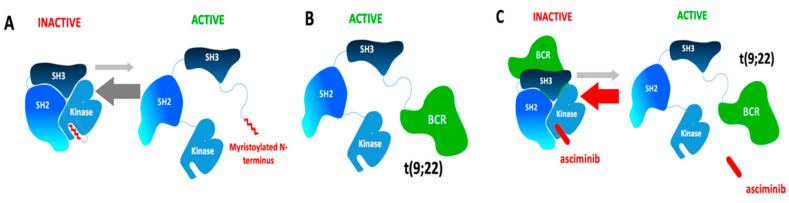
Binding of the Myristoyl Site of the BCR-ABL1 Protein by Asciminib. Autoinhibition of the ABL1 kinase occurs through engagement of the myristoyl-binding site by the myristoylated *N*-terminal-a negative regulatory motif that locks the ABL1 kinase in the inactive state (**A**). On fusion of ABL1 to BCR, the myristoylated *N*-terminal is lost and de ABL1 kinase is activated (**B**). By allosterically binding the myristoyl site, asciminib mimics myristate and restores inhibition of BCR-ABL1 kinase activity (**C**).

Asciminib is a new BCR-ABL1 inhibitor that differs from previous TKIs in that it does not bind to the ATP-binding site of the kinase. Rather, asciminib acts as an allosteric inhibitor targeting the vacant site of the pocket commonly occupied by the myristoylated N-terminus of ABL1 (a motif that serves as an allosteric negative regulatory element loss of fusion of ABL1 to BCR; Figure 3). Asciminib is highly selective for ABL1 and ABL2 kinases and inhibits proliferation of unmutated BCR-ABL1 and ATP site mutants including T315I [32].

Due to this new mechanism of action, asciminib offers the possibility of dual inhibition of BCR-ABL1 in combination with ATP-binding TKIs. These combinations could potentially improve results by providing enhanced target coverage [33].

**Figure 4:**
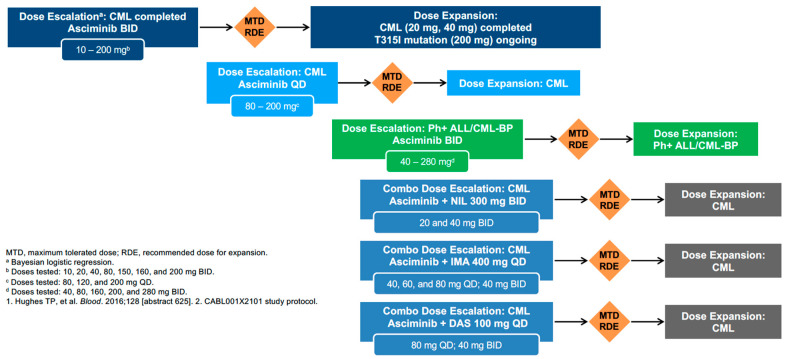
Phase 1 FIH study: single agent in CML/ALL and combinations in combinations in CML (CABL001 × 2101). NIL = nilotinib; BID = twice a day; QD = once a day; MTD: maximum tolerated dose; RDE: recommended dose for expansion.

A phase I study was conducted to determine the safety and efficacy of asciminib in patients with Ph-positive leukemia after failure of prior TKIs in different clinical situations, including mutational status or disease phase [34]. This study also evaluated asciminib in monotherapy or combined with imatinib, nilotinib, or dasatinib (Figure 4).

#### 3.1.1. Asciminib Monotherapy

Results of asciminib monotherapy in CML patients in CP (*n* = 141) or accelerated phase (AP; *n* = 9) have recently been published (Table 3). Patients were heavily pretreated, 70% having received at least three previous TKIs (including ponatinib in 40% of cases). Of interest, 31% of patients harbored at least one BCR-ABL1 kinase domain mutation, the most frequent of which was the 315I mutation. Maximum tolerated dose was not reached and recommended dose for phase II trials was established at 40 mg BID after 5 dose-limiting toxic effects.

Asciminib was well tolerated, with the most common nonhematologic adverse events being grade 1 or 2 asymptomatic lipase and/or amylase elevation, rash, and constitutional symptoms. Five patients (3%) developed clinical pancreatitis (all were receiving >40 mg BID and three had prior history of pancreatitis with other TKIs). Myelosuppression was uncommon and mostly grade 1/2.

Efficacy results were given based on presence of the T315I mutation. In patients without T315I mutation, 92% and 54% of patients without complete hematological response (CHR) and CCyR at baseline achieved these responses, respectively. MMR was achieved or maintained in 48% of patients. Of note, the probability of achieving MMR was highly dependent on baseline status. Thus, MMR rates of 75% vs. 27% were achieved in patients with BCR-ABL1 transcript levels below or above 1% at baseline, respectively. Overall, degree of molecular response improved in 63% of patients.

Among 28 patients harboring the T315I mutation, 88%, 41%, and 24% achieved CHR, CCyR, and MMR, respectively. Only one patient out of five previously treated with ponatinib obtained MMR. Of interest, three out of four patients achieving MMR received doses above 150 mg BID [23].

Data have recently been presented on a dose expansion cohort of 32 patients with T315I mutation treated with asciminib 200 mg BID. A total of 37% and 21% had previous resistance and intolerance to ponatinib, respectively. Asciminib was generally well tolerated, with only one patient discontinuing treatment due to adverse events. In terms of efficacy, the probability of obtaining or maintaining CCgR and MMR after a median exposure of 27 weeks was 66% and 36%, respectively. In line with previous results, efficacy depended greatly on prior ponatinib treatment, with 61% and 17% MMR rates in patients without and with prior ponatinib exposure, respectively [35].

#### 3.1.2. Asciminib Combo Trials

Phase I studies included combination dose escalation sub-studies where asciminib at different doses was combined with imatinib, nilotinib or dasatinib.

Asciminib in combination with imatinib:

Dr Cortes et al. [36] presented data from 25 heavily pretreated patients receiving asciminib at different dose levels (40, 60, 80 mg QD or 40 mg BID) in combination with imatinib 400 mg QD. A total of 15 patients (60%) had received more than two prior TKIs and 17 (68%) had received prior imatinib. At the time of data cut-off, treatment was ongoing in 17 patients (68%). Reasons for discontinuation were patient/physician decision (*n* = 4), adverse events (pruritus (*n* = 1) and proximal myopathy (*n* = 1)), pregnancy (*n* = 1), and progressive disease (*n* = 1). Median treatment exposure was 54.6 weeks. Grade 3/4 adverse events were observed in 11 patients (44%); most common any-grade adverse events were nausea (32%), increased lipase (20%) and abdominal pain, peripheral edema, and vomiting (16% each). In terms of efficacy, for patients with BCR-ABL1IS <1% at baseline, 9/15 (60%) maintained this degree of response by 48 weeks. Among evaluable patients without MMR at baseline, 8/19 (42%) achieved MMR [36].

Asciminib in combination with dasatinib or nilotinib:

New results have become available on 34 patients treated with a combination of asciminib with dasatinib or nilotinib, in which the asciminib dose differed depending on the combination (asciminib 20 mg or 40 mg BID with nilotinib 300 mg BID and asciminib 40 mg or 80 mg BID with dasatinib 100 mg). In general, all combinations were safe and well tolerated. One dose-limiting toxicity was reported with asciminib plus nilotinib (maculopapular rash) and one with asciminib plus dasatinib (increased lipase levels); both events resolved with dose reduction. Only one patient discontinued treatment due to adverse events in each combination. One patient with a history of cardiovascular disease receiving asciminib 40 mg BID in combination with nilotinib developed peripheral arterial occlusive disease (PAOD). Pleural effusion was reported in two patients with asciminib combined with nilotinib (both had received dasatinib just prior to study entry) and in two patients treated with asciminib in combination with dasatinib. There were no cases of clinical pancreatitis or on-treatment deaths with either combination. In terms of efficacy, among patients without BCR-ABL1IS <1% at baseline, 6/14 (43%) and 5/9 (56%) patients treated with asciminib plus nilotinib or dasatinib, respectively, achieved this response by 48 weeks. In patients without MMR at baseline, 4/13 (31%) and 5/14 (36%) patients on asciminib plus nilotinib or dasatinib combinations, respectively, achieved MMR by 48 weeks. No patient with baseline MMR lost this degree of response [37].

Clinical trials evaluating asciminib in different scenarios are currently ongoing. The ASCEMBL trial (NCT03106779) randomized 222 CP CML patients that had previously failed at least two TKIs to receive asciminib 40 mg BID or bosutinib 500 mg daily. Enrolment is complete and results are pending presentation. The primary endpoint of the study was achievement of MMR. If more favorable results are reached in the asciminib arm, this drug could presumably gain approval for third-line treatment in CML CP patients. The ASC4MORE trial (NCT0357836) is evaluating use of asciminib in combination with nilotinib to improve molecular response in patients without optimal response to imatinib.

Finally, future directions are promising with regards to the potential efficacy of combination treatment of asciminib with ponatinib in patients with TKI refractory CML harboring BCR-ABL1 compound mutants [38].

### 3.2. HQP1351

HQP1351 is a novel, orally active potent third-generation TKI designed for treatment of CML patients with resistance to current TKI therapies including those with T315I mutation [39]. The Food and Drug Administration (FDA) has granted orphan drug designation to HQP1351 as treatment for CML patients based on results from a phase I study conducted in China. A total of 101 CP or AP CML patients with resistance or intolerance to ≥2 prior TKIs or with T315I mutation after ≥1 prior TKI were included. After a median follow-up of 12.8 months, MCgR and CCyR rates in resistant patients were 82% and 78%, respectively. Newly achieved CCyR and MMR were observed in 69% and 37% of CP CML patients, respectively. The drug was well tolerated at all three dose levels. The most common grade 3/4 treatment-related adverse event (TRAE) was thrombocytopenia, occurring in 50% of patients [40].

### 3.3. PF-114

PF-114, a derivative of the third-generation BCR-ABL inhibitor ponatinib, has demonstrated strong inhibitory activity against wild-type and mutant BCR-ABL forms, such as the clinically important T315I. Furthermore, PF-114 has exhibited preferential kinase selectivity, favorable safety, notable pharmacokinetic properties, and therapeutic efficacy in a murine model [41,42].

Data from a phase I study in subjects with CP or AP CML failing ≥2 TKIs or who have BCR-ABL1 T315I mutation (NCT02885766) showed achievement of MCyR in 6/21 patients (most responses were observed at daily doses of 200 mg and 300 mg). Skin toxicity (psoriasis-like skin lesions) with doses above 400 mg was the most common non-hematology toxicity (resolving rapidly after treatment discontinuation) [43]. An ongoing phase II clinical trial is evaluating PF-114 in CML patients resistant to previous TKIs.

### 3.4. K0706

K0706 is a novel third-generation TKI effective against wild-type and mutated BCR-ABL1 isoforms with significantly less off-target activity than existing TKIs. Reports from the phase I clinical trial of K0706 in patients who were resistant and/or intolerant to ≥3 prior TKIs or who had co-morbidities precluding the use of 2GTKI (NCT02629692) have recently been presented [44]. K0706 showed good tolerability with mild to moderate gastrointestinal disturbance (18%) and fatigue (15%). K0706 showed preliminarily good clinical efficacy with a high proportion of patients achieving CCyR in heavily pretreated CP CML patients. The drug also showed efficacy in advanced phases [45]. K0706 development is ongoing in a phase I/II clinical trial.

## 4. General Considerations

Despite the excellent prognosis of CML patients in terms of OS, 2GTKI treatment failure is a complication that physicians frequently face in clinical practice. Clinical trials with nilotinib, dasatinib, or bosutinib as second-line therapy have shown that around 70% of patients discontinue treatment after 4 years of follow-up. An Italian research group has recently reported on outcomes in CML patients treated with front-line 2GTKI in clinical practice. Of note, only 13% of patients required treatment change due to resistance (42%), intolerance (32%), or other reasons (16%) after a median follow-up of 12 months. These findings differ from previous data from clinical trials in which treatment discontinuation rates were much higher. In this regard, the authors speculate that the main reason for such a difference could relate to the younger age of patients receiving upfront 2GTKI treatment compared to industry-sponsored clinical trials [46]. There are no studies comparing treatment responses between patients failing 2GTKI as front- or later-lines. Breccia et al. published the experience of patients treated with ponatinib in second-line after failed first-line 2GTKI. Overall, results were better compared to patients treated in third- or later-lines, with the caveat that the majority of patients were treated due to intolerance, in contrast to later-line studies in which most patients were resistant to 2GTKI [27].

When addressing 2GTKI treatment failure there are several important considerations. Failure is an operational concept, defining patients who no longer benefit from a particular drug due either to lack of efficacy or side effects. The four approved drugs currently available in this setting differ in terms of efficacy and tolerability [2]. Given this, it is crucial to pinpoint the specific reason for drug failure and to identify patient comorbidities and history of side effects with prior TKIs in order to prescribe the optimal treatment option for each particular patient.

Current European Leukemia Net (ELN) recommendations define resistance as failing to achieve BCR-ABL1 ≤ 1% (or CCyR) at 12 months of front-line or second-line treatment. Intolerance is usually defined by appearance of grade 3/4 side effects, although responding patients with grade 2 chronic toxicity may be willing to change therapy. However, there are situations in which intolerance and failure may overlap, for instance, in patients who need dose modifications due to persistent side effects that compromise response can be difficult to classify [2].

National Comprehensive Cancer Network (NCCN) and European Society for Mediacal Oncology (ESMO) guidelines recommend any 2GTKI and ponatinib at the same level to manage patients with inadequate response to 2GTKI. The main factors to take into account for drug selection are side effects with prior TKIs and mutational status [47,48]. On the other hand, ELN recommendations support preferential selection of ponatinib for 2GTKI resistant patients [2]. Although no head-to-head comparison has been made between ponatinib and other 2GTKI in this clinical situation, available data from single agent clinical trials and real-world studies support ponatinib use in 2GTKI resistant patients, due to the higher probability of achieving optimal response with this drug. It should be underlined that efficacy of ponatinib is influenced by the number of previous lines of therapy and undue delay in treatment initiation should therefore be avoided. [2,13,24]. However, it is important to consider the potentially severe side effects of ponatinib, especially in patients with concomitant cardiovascular risk factors. In this sense, current evidence shows a strong relationship between previous cardiovascular risk factors and occurrence of occlusive vascular events while on ponatinib [26]. Preliminary results from the OPTIC trial showed a trend toward dose-dependent efficacy and safety that may provide a better understanding of the ponatinib risk/benefit profile. Pending the final results from the study, there is as yet no consensus on the best starting dose. A group of ELN panel experts agreed that ponatinib dosing should be determined according to assessment of expected risk–benefit based on CML characteristics, BCR-ABL1 mutational status, and patients’ cardiovascular risk. In CP CML patients, a 30 mg/day dose was considered the preferred option even in patients with low cardiovascular risk [49]. In contrast, experts from a German consensus group coincided on a further decrease in ponatinib starting dose for CP CML patients failing single TKI with good baseline response (at least MCyR), absence of mutations, history of TKI intolerance rather than resistance, and a high-risk cardiovascular profile [50]. Finally, a recent update from the ELN expert panel recommends a lower daily starting dose of 15 mg or 30 mg in patients with lesser degrees of TKI resistance or multiple intolerances, especially in case of increased cardiovascular risk profile. The panel recommends prescribing the 45 mg daily dose in patients with T315I or compound mutations, or progressing to advanced CML phase [2]. The benefits of aspirin use to avoid occlusive disease remain unclear [24,26].

In 2GTKI resistant patients with high cardiovascular risk profile, alternative 2GTKIs should be considered. A definition of high cardiovascular risk in the setting of TKI treatment for CML has not yet been standardized. Data from the Italian CML group supports using the Social Cohesion and Reconciliation (SCORE) index to identify ponatinib treated patients with a higher risk of cardiovascular complications [26].

Around a fourth of 2GTKI resistant patients can achieve optimal responses with bosutinib treatment. Higher response rates have been reported in the recent BYOND study; however, differences in treatment response assessment and study populations must be taken into consideration when comparing results from these studies [16,18].

Intolerance to 2GTKI can be approached by careful assessment of prior side effects and proper knowledge of the toxicity profile of each 2GTKI. Recent data from the BYOND study showed impressive results in terms of efficacy and safety in patients with intolerance to prior TKIs. Importantly, data from the BYOND study as well as retrospective studies suggest that lower bosutinib doses (300–400 mg) could be adequate to manage 2GTKI intolerant patients [17,51].

A subset of resistant/intolerant CML patients cannot be rescued with currently approved treatment options and new drugs are therefore warranted. Asciminib has shown striking efficacy in heavily pretreated patients. Remarkably, 57% of asciminib treated patients with previous resistance or intolerance to ponatinib achieved or maintained MMR in a recently published trial. In patients harboring T315I mutations, treatment responses were influenced by previous ponatinib exposure. Overall, the safety profile of asciminib is very favorable, as predicted by the high selectivity for BCR-ABL1 binding due to its unique mechanism of action [33]. Nevertheless, longer follow-up and clinical data from more patients treated under the compassionate use program are necessary to confirm its safety profile. Results from the ASCEMBL trial will be valuable to define the efficacy of asciminib in patients failing 2GTKIs, although a formal comparison with ponatinib in this setting is still warranted.

**Figure 5:**
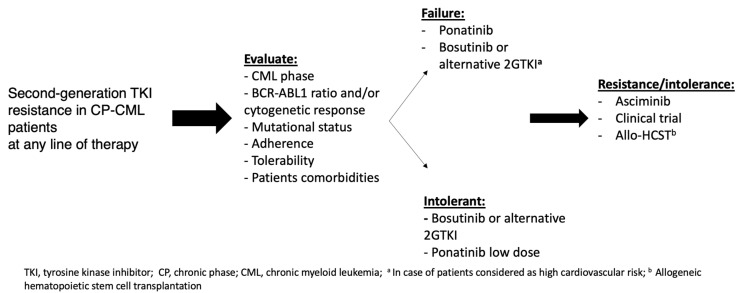
Treatment approach in CML patients failing 2GTKI. *Allo-HCST* = allogenic hematopoietic stem cell transplantation; *2GTKI* = second generation tyrosin kinase inhibitor.

In conclusion. At the present time, several therapeutic options for patients failing 2GTKIs are available. Therefore, the choice of treatment should be based on aspects related treatment response, side effects suffered with previous TKIs and patients’ comorbidities (Figure 5).

## Figures and Tables

**Table 1:** Summary of adverse events in the BYOND study.

	Total *n* = 163	
TEAE, † *n* (%)	All Grades	Grade 3/4
Any TEAE	162 (99)	120 (73)
Diarrhea	143 (87)	26 (16)
Nausea	65 (39)	4 (2)
Vomiting	53 (32)	6 (3)
Abdominal Pain	46 (28)	7 (4)
Headache	45 (27)	1 (1)
Elevated ALT	42 (25)	23 (14)
Fatigue	39 (23)	2 (1)
Upper abdominal Pain	36 (22)	2 (1)
Dyspnea	35 (21)	5 3)
Asthenia	33 (20)	4 (2)

* The safety population was defined as all-grade TEAEs reported in >20% of patients. † Classification of adverse events is based on the Medical Dictionary for Regulatory Activities (version 21.1). ALT = alanine aminotransferase; TEAE = treatment-emergent adverse event.

**Table 2:** Ponatinib studies in clinical practice.

	Heiblig et al. [24]	Shacham-Abulafia et al. [25]	Caocci et al. [26]	Breccia [27]	Chan et al. [28]
	48 (CP = 6 AP) 8 (BP)	37 (21 CP, 6 AP, 10 BP)	86 CP	29 CP	78 (51 CP, 9 AP, 18 PB)
Previous TKIs >2 >3	27% 66%	24% 52%	43% 29%	100% treated 2L	33% 65%
Age at Ponatinib Initiation (years)	60	43	53	55	42
T315I Mutation	25%	20%	24%	7%	20%
Previous Occlusive Vascular Events	24%	0%	4%	0%	NS
Ponatinib Starting Dose (mg)	Median 45 mg (range 15–45)	45 mg: 60% 30 mg: 32%; 15 mg: (8%)	45 mg: 40% 30 mg: 42% 15 mg:18%	45 mg: 60% 30 mg: 38% 15 mg: 2%	Median 39 mg
Median Follow up (Months)	26	14	28	12	40
CCyR in CP CML Population	64% (6 months)	61% (6 months)	NA	59%	69% (any time)
Vascular Occlusive Events	17%	3%	16%	0	23%

CML = Chronic myeloid leukemia; P = chronic phase; AP = accelerated phase; BP = blastic phase; 2L = second-line; CCyR = complete cytogenetic response; NS = not shown.

**Table 3:** Responses in asciminib treated patients.

Chronic-Phase CML						
	No T315 mMutation			T315I Mutation		
	Overall (*n* = 113)	Response Achieved	Response Maintained	Overall (*n* = 28)	Response Achieved	Response Maintained
Pts Remaining in Study-*n* (%)	88 (78%)			19 (68%)		
CHR		34/37 (92%)			14/16 (88%)	
MCyR	85/110 (77%)	24/40 (60%)	61/70 (87%)	15/25 (60%)	11/20 (55%)	4/5 (80%)
CCyR	77/110 (70%)	31/57 (54%)	46/53 (87%)	11/25 (44%)	9/22 (41%)	2/3 (67%)
MMR	44/91 (48%)	26/72 (36%)	18/19 (95%)	5/18 (28%)	4/17 (24%)	1/1 (100%)

*Pts = patients; n* = number of patients; 2L = second-line; CHR = complete hematological response; CCyR = complete cytogenetic response; MMR = major molecular response.
